# Non–SARS-CoV-2 Respiratory Viruses in Athletes at Major Winter Sport Events, 2021 and 2022

**DOI:** 10.3201/eid2810.220478

**Published:** 2022-10

**Authors:** Maarit Valtonen, Matti Waris, Raakel Luoto, Katja Mjøsund, Mira Kaikkonen, Olli J. Heinonen, Olli Ruuskanen

**Affiliations:** Finnish Institute of High Performance Sport KIHU, Jyväskylä, Finland (M. Valtonen¸ M. Kaikkonen);; University of Turku and Turku University Hospital, Turku, Finland (M. Waris, R. Luoto, O. Ruuskanen);; University of Turku, Turku, Finland (K. Mjøsund, O.J. Heinonen)

**Keywords:** respiratory infections, respiratory viruses, athletes, COVID-19, rhinovirus, common cold, coronavirus disease, SARS-CoV-2, severe acute respiratory syndrome coronavirus 2, viruses, winter sports, sport events, Olympics, Finland

## Abstract

We performed prospective studies on respiratory viral infections among Team Finland participants during the 2021 Oberstdorf World Ski Championships and the 2022 Beijing Olympic Games. We enrolled 73 athletes and 110 staff members. Compared with similar studies we conducted before the COVID-19 pandemic, illnesses and virus detections dropped by 10-fold.

Elite athletes have an increased risk for contracting acute respiratory infections (ARIs) during major winter sport events (*1*–*5*), which often occur during viral peaks in the community. Many of the athletes’ behavioral factors during events, such as using public transportation, crowding, using group accommodation, and close socializing activities, may all increase an athlete’s susceptibility to acute respiratory viral infection (*6*).

The aim of our study was to investigate the occurrence of respiratory viruses in sport teams during 2 major winter sport events that implemented public and individual COVID-19 prevention procedures. For comparison, we used observations from 2 previous studies in corresponding competitions conducted before the COVID-19 pandemic (*3*,*5*).

## The Study

We conducted the studies at the Nordic World Ski Championships in Oberstdorf, Germany, during February 18–March 7, 2021, and at the Olympic Winter Games in Beijing, China, during January 27–February 21, 2022. In Oberstdorf, 633 athletes from 65 countries participated, and in Beijing, 2,871 athletes from 91 countries participated. Our study included 73 Team Finland members in Oberstdorf (26 athletes and 47 staff) and 110 in Beijing (47 athletes and 63 staff); we excluded the ice hockey teams. We monitored team members for the duration of their trip, starting from their arrival at the event location and finishing with their departure from the hotel. At arrival at both events, all team members were asymptomatic. ARI (i.e., the common cold) was defined as the acute onset of any of the following signs and symptoms: sore throat, rhinorrhea, nasal congestion, hoarseness, cough, and fever (>37.8°C) (*7*).

In addition to the SARS-CoV-2 testing conducted by the event organizers (every other day in Oberstdorf and every day in Beijing), we collected flocked nasal swab specimens from the team in Oberstdorf on days 1, 7, and 13 with minor variations. In Beijing, nasal swab specimens were taken only from participants with acute onset of a respiratory symptom or symptoms.

We conducted all study-related activities according to Guideline for Good Clinical Practice (https://www.ema.europa.eu/en/documents/scientific-guideline/ich-e-6-r2-guideline-good-clinical-practice-step-5_en.pdf), which includes the provisions in the Declaration of Helsinki. The protocol was approved by the Ethics Committee of the Hospital District of Southwest Finland (Oberstdorf) and the Ethics Committee, Central Finland Health Care District (Beijing).

COVID-19 countermeasures included relative quarantine of the team members before traveling (i.e., use of masks and physical distancing); having a negative SARS-CoV-2 test before departure; using masks during travel and at the games; traveling by chartered flights; following enhanced hand hygiene and environmental disinfection; maintaining physical distance; housing in single or double rooms; limiting use of indoor public facilities; and allowing no spectators in Oberstdorf and a limited number in Beijing. The team in Beijing was fully vaccinated against COVID-19; the team in Oberstdorf was unvaccinated.

We performed laboratory testing at the site in Beijing using a BioFire FilmArray Respiratory Panel 2.1 Plus (BioFire, https://www.biofiredx.com). The panel detects the following viruses: respiratory syncytial virus; adenovirus; influenza A and B viruses; rhinovirus/enterovirus; parainfluenza type 1–4 viruses; human coronaviruses 229E, OC43, HKU1, and NL63; SARS-CoV-2; Middle East respiratory syndrome coronavirus; and human metapneumovirus. We retrospectively tested the Oberstdorf samples in Turku, Finland, using Allplex Respiratory Panels 1–3 (Seegene, https://www.seegene.com). This panel detects 16 viruses that are otherwise the same as FilmArray except that it differentiates between rhinoviruses and enteroviruses and detects human bocavirus but does not detect coronavirus HKU1, SARS-CoV-2, and MERS-CoV. Furthermore, we tested these samples with a laboratory-designed PCR for rhinoviruses, enteroviruses, and respiratory syncytial virus (*8*). The event organizers screened for SARS-CoV-2 using PCR assays in Oberstdorf and in Beijing.

In Oberstdorf, no cases of symptomatic ARIs were verified among the 73 sport team members. All 357 PCR tests for SARS-CoV-2 performed by the organizers were negative. Of a total of 203 nasal mucus samples, we detected rhinovirus in 2 samples, both collected on day 1 of the event from 1 athlete and 1 staff member.

At arrival at the airport in Beijing, 1 asymptomatic athlete tested positive for SARS-CoV-2. However, retesting in the Olympic Village proved negative. We recorded 6 cases of symptomatic ARIs, and we identified a virus in 4 of them. We detected 1 respiratory syncytial virus in 1 athlete on day 1, 1 metapneumovirus in 1 staff member on day 2, and 1 coronavirus 229E in 1 athlete on day 3. In 1 staff member, an ARI was evident on return to Finland and was identified as coronavirus OC43.

## Conclusions

We found only 6 cases of symptomatic ARI among 183 (3%) members of Team Finland during 2 major winter sports events (the 2021 World Ski Championships and the 2022 Beijing Olympic Winter Games). The difference between these events and the historical comparison groups before COVID-19 is dramatic. At the January 26–February 28, 2018, Olympic Winter Games in PyeongChang, South Korea, and the February 18–March 3, 2019, World Ski Championships in Seefeld, Austria, ARIs were recorded in 58 (33%) of the 174 members of Team Finland (*3*,*5*) (Table). Clinically, all the ARIs were mild common colds.

**Table:** ARIs among Team Finland during 4 major winter sport events before and during the COVID-19 pandemic, 2018–2022*

Event	2022 Winter Olympic Games	2021 Nordic World Ski Championships	2019 Nordic World Ski Championships	2018 Winter Olympic Games
Location	Beijing, China	Oberstdorf, Germany	Seefeld, Austria	PyeongChang, South Korea
Study period	Jan 27–Feb 21	Feb 18–Mar 7	Feb 18–Mar 3	Jan 26–Feb 28
Median length of stay, d	21	14	14	21
Local viral season	Not known	Low	Medium/high	High
Team members, no.	110	73	62	112
Athletes	47	26	26	44
Staff	63	47	36	68
ARIs, no. (%)	6 (5)	0	16 (26)	42 (38)
Athletes	3 (6)	0	10 (38)	20 (45)
Staff	3 (5)	0	6 (17)	22 (32)
Virus detections,† no. (%)	4 (67)	0	14 (90)	30 (71)
Athletes	2 (67)	0	8 (80)	15 (75)
Staff	2 (67)	0	6 (100)	15 (68)
Asymptomatic persons tested,† no. (no. tests)	0	73 (203 tests)‡	62 (158 tests)‡	34 (34 tests)§
Virus detections	0	2 (3)	10 (16)	6 (18)
SARS-CoV-2 testing#				
Total no. tests at event	≈1,800,000	≈20,000	NA	NA
No. positive	437	9	NA	NA
Reference	This study	This study	(*3*)	(*5*)

*ARI, acute respiratory infection; NA, not applicable. †Analyses of the study for other than SARS-CoV-2 respiratory viruses. ‡Active surveillance of asymptomatic infections. §Only high-risk contacts of symptomatic case-patients were tested. #Surveillance conducted by event organizers.

In the Oberstdorf and Beijing winter sport events, we detected only 3 (4%) non–SARS-CoV-2 infections (caused by 3 different viruses) in 73 athletes, and those infections did not spread further. Symptom onset was 1–3 days after arrival in Beijing, which suggests that the infections were acquired in Finland. We recorded no SARS-CoV-2–positive results among Team Finland at either event, although COVID-19 cases were detected among other teams at both events (Table). In contrast, in our 2 earlier studies, conducted amid limited mitigation strategies (e.g., enhanced hand hygiene and disinfection and isolation of symptomatic persons), we detected respiratory viral infections (caused by 9 different viruses) in 30 (50%) of the 60 athletes (Table). Those infections spread readily among the team members (*3*,*5*,*6*).

Control measures during the COVID-19 pandemic markedly reduced the global occurrence of non–SARS-CoV-2 respiratory viruses (*9*–*12*). For example, a 98%–99% decrease in the detection of respiratory syncytial and influenza virus infections throughout the winter of 2020 was reported in Australia (*11*). During the 2021 Championships in Obertsdorf, low prevalences of only rhinoviruses and seasonal coronaviruses (no influenza) were observed in the community (https://www.rki.de/EN/Home/homepage.html). Furthermore, no or only a few spectators attended the events. Minimal environmental viral pressure is most probably the major explanation for our observations, and individual prevention procedures inhibited transmission of detected viruses among the team.

Limitations of our study include the fact that the number of team members was low and the methods for viral diagnostics varied, but neither of these factors should affect the overall results. We carefully recorded occurrence of ARIs in each study, but comprehensive testing of all participants for asymptomatic infections might have increased the numbers of viruses detected in some of the studies.

In summary, our observations suggest that multilayered mitigation strategies effectively prevented respiratory viral infections during 2 major winter sport events that occurred during the COVID-19 pandemic. Sport events may be held without an increased risk for respiratory viral infections (*13*). ARIs are now returning, concurrent with relaxed control measures (*14*,*15*). It remains to be clarified what mitigation procedures will be sufficiently effective in preventing respiratory viral infections during major sports events after the COVID-19 pandemic while, at the same time, minimally affecting the well-being of the athletes.

## References

[1] Gundlapalli AV, Rubin MA, Samore MH, Lopansri B, Lahey T, McGuire HL, et al. Influenza, winter Olympiad, 2002. Emerg Infect Dis. 2006;12:144–6. 10.3201/eid1201.050645 16494733 PMC3291389

[2] Svendsen IS, Gleeson M, Haugen TA, Tønnessen E. Effect of an intense period of competition on race performance and self-reported illness in elite cross-country skiers. Scand J Med Sci Sports. 2015;25:846–53. 10.1111/sms.12452 25818900

[3] Valtonen M, Waris M, Vuorinen T, Eerola E, Hakanen AJ, Mjøsund K, et al. Common cold in Team Finland during 2018 Winter Olympic Games (PyeongChang): epidemiology, diagnosis including molecular point-of-care testing (POCT) and treatment. Br J Sports Med. 2019;53:1093–8. 10.1136/bjsports-2018-100487 31142472 PMC6818521

[4] Nabhan D, Windt J, Taylor D, Moreau W. Close encounters of the US kind: illness and injury among US athletes at the PyeongChang 2018 Winter Olympic Games. Br J Sports Med. 2020;54:997–1002. 10.1136/bjsports-2018-100015 31375500

[5] Valtonen M, Grönroos W, Luoto R, Waris M, Uhari M, Heinonen OJ, et al. Increased risk of respiratory viral infections in elite athletes: A controlled study. PLoS One. 2021;16:e0250907. 10.1371/journal.pone.0250907 33945550 PMC8096105

[6] Ruuskanen O, Luoto R, Valtonen M, Heinonen OJ, Waris M. Respiratory viral infections in athletes: many unanswered questions. Sports Med. 2022;52:2013–21. 10.1007/s40279-022-01660-9 35353361 PMC8965548

[7] Puhakka T, Mäkelä MJ, Malmström K, Uhari M, Savolainen J, Terho EO, et al. The common cold: effects of intranasal fluticasone propionate treatment. J Allergy Clin Immunol. 1998;101:726–31. 10.1016/S0091-6749(98)70301-X 9648698

[8] Österback R, Tevaluoto T, Ylinen T, Peltola V, Susi P, Hyypiä T, et al. Simultaneous detection and differentiation of human rhino- and enteroviruses in clinical specimens by real-time PCR with locked nucleic Acid probes. J Clin Microbiol. 2013;51:3960–7. 10.1128/JCM.01646-13 24048533 PMC3838063

[9] Groves HE, Piché-Renaud PP, Peci A, Farrar DS, Buckrell S, Bancej C, et al. The impact of the COVID-19 pandemic on influenza, respiratory syncytial virus, and other seasonal respiratory virus circulation in Canada: A population-based study. Lancet Reg Health Am. 2021;1:100015. 10.1016/j.lana.2021.100015 34386788 PMC8285668

[10] Olsen SJ, Winn AK, Budd AP, Prill MM, Steel J, Midgley CM, et al. Changes in influenza and other respiratory virus activity during the COVID-19 pandemic—United States, 2020–2021. MMWR Morb Mortal Wkly Rep. 2021;70:1013–9. 10.15585/mmwr.mm7029a1 34292924 PMC8297694

[11] Yeoh DK, Foley DA, Minney-Smith CA, Martin AC, Mace AO, Sikazwe CT, et al. Impact of coronavirus disease 2019 public health measures on detection of influenza and respiratory syncytial virus in children during the 2020 Australian winter. Clin Infect Dis. 2021;72:2199–202. 10.1093/cid/ciaa1475 32986804 PMC7543326

[12] Oh DY, Buda S, Biere B, Reiche J, Schlosser F, Duwe S, et al. Trends in respiratory virus circulation following COVID-19-targeted nonpharmaceutical interventions in Germany, January - September 2020: Analysis of national surveillance data. Lancet Reg Health Eur. 2021;6:100112. 10.1016/j.lanepe.2021.100112 34124707 PMC8183189

[13] Schultz EA, Kussman A, Jerome A, Abrams GD, Hwang CE. Comparison of SARS-CoV-2 test positivity in NCAA Division I student athletes vs nonathletes at 12 institutions. JAMA Netw Open. 2022;5:e2147805. 10.1001/jamanetworkopen.2021.47805 35138397 PMC8829663

[14] Chan HC, Tambyah PA, Tee NWS, Somani J. Return of other respiratory viruses despite the disappearance of influenza during COVID-19 control measures in Singapore. J Clin Virol. 2021;144:104992. 10.1016/j.jcv.2021.104992 34619380 PMC8485711

[15] Haapanen M, Renko M, Artama M, Kuitunen I. The impact of the lockdown and the re-opening of schools and day cares on the epidemiology of SARS-CoV-2 and other respiratory infections in children - A nationwide register study in Finland. EClinicalMedicine. 2021;34:100807. 10.1016/j.eclinm.2021.100807 33817612 PMC8007090

